# Physical Function and Physical Activity in Older Breast Cancer Survivors: 5-Year Follow-Up from the Climb Every Mountain Study

**DOI:** 10.1093/oncolo/oyad027

**Published:** 2023-03-21

**Authors:** Annelieke A Lemij, Gerrit Jan Liefers, Marloes G M Derks, Esther Bastiaannet, Marta Fiocco, Titia E Lans, Carmen C van der Pol, Annelie J E Vulink, Leander van Gerven, Onno R Guicherit, Eugenie M H Linthorst-Niers, Jos W S Merkus, Thijs van Dalen, Johanneke E A Portielje, Nienke A de Glas

**Affiliations:** Department of Medical Oncology, Leiden University Medical Center, Leiden, The Netherlands; Department of Surgery, Leiden University Medical Center, Leiden, The Netherlands; Department of Surgery, Leiden University Medical Center, Leiden, The Netherlands; Department of Medical Oncology, Leiden University Medical Center, Leiden, The Netherlands; Department of Medical Oncology, Leiden University Medical Center, Leiden, The Netherlands; Department of Surgery, Leiden University Medical Center, Leiden, The Netherlands; Mathematical Institute, Leiden University and Department of Biomedical Data Science, Section Medical Statistics, Leiden University Medical Center, Leiden, The Netherlands; Department of Surgery, Admiraal de Ruyter Hospital, Goes, The Netherlands; Department of Surgery, Alrijne Hospital, Leiden and Leiderdorp, The Netherlands; Department of Medical Oncology, Reinier de Graaf Gasthuis, Delft, The Netherlands; Department of Internal Medicine, LangeLand Hospital, Zoetermeer, The Netherlands; Department of Surgery, Haaglanden Medical Center, The Hague, The Netherlands; Department of Surgery, Groene Hart Hospital, Gouda, The Netherlands; Department of Surgery, Haga Hospital, The Hague, The Netherlands; Department of Surgery, Diakonessenhuis, Utrecht, The Netherlands; Department of Medical Oncology, Leiden University Medical Center, Leiden, The Netherlands; Department of Medical Oncology, Leiden University Medical Center, Leiden, The Netherlands

**Keywords:** breast cancer, physical activity, functional status, older patients, geriatric assessment

## Abstract

**Background:**

A decline in physical activity and the ability to perform activities of daily living (ADL) and instrumental activities of daily living (IADL) could interfere with independent living and quality of life in older patients, but may be prevented with tailored interventions. The aim of the current study was to assess changes in physical activity and ADL/IADL in the first 5 years after breast cancer diagnosis in a real-world cohort of older patients and to identify factors associated with physical decline.

**Methods:**

Patients aged ≥70 years with in situ or stages I-III breast cancer were included in the prospective Climb Every Mountain cohort study. Linear mixed models were used to assess physical activity (according to Metabolic Equivalent of Task (MET) hours per week) and ADL/IADL (according to the Groningen Activity Restriction Scale (GARS)) over time. Secondly, the association with geriatric characteristics, treatment, quality of life, depression, apathy, and loneliness was analyzed.

**Results:**

A total of 239 patients were included. Physical activity and ADL/IADL changed in the first 5 years after diagnosis (mean change from baseline −11.6 and +4.2, respectively). Geriatric characteristics at baseline were strongly associated with longitudinal change in physical activity and ADL/IADL, whereas breast cancer treatment was not. A better quality of life was associated with better physical activity and preservation of ADL/IADL, while depression and loneliness were negatively associated with these outcomes.

**Discussion:**

Geriatric characteristics, loneliness, and depressive symptoms were associated with physical decline in older patients with breast cancer, while breast cancer treatment was not.

## Teaser

A decline in physical activity and the ability to perform activities of daily living may interfere with quality of life in older patients. This article assessed changes in physical activity and activities of daily living in the first 5 years after breast cancer diagnosis in a real-world cohort of older patients and identified factors associated with physical decline.

**Implications for Practice**

Older patients may value maintenance of independence and quality of life over longevity. Although >30% of new breast cancer cases occur in patients aged 70 years or older, little is known about these outcomes for this age group. This study showed that physical activity and the ability to carry out daily tasks decreased in the first 5 years after diagnosis. These changes did not seem to be related to breast cancer or its treatment, but rather to pre-existent geriatric characteristics, loneliness, and depressive symptoms. Greater awareness of older patients’ heterogeneity and its implications is needed to improve individualized care for the older population.

## Introduction

Breast cancer is the most commonly diagnosed malignancy among women, with more than 30% of patients being over 70 years of age at the time of diagnosis.^1^ This proportion is expected to increase due to the rapidly aging population. The older population is a heterogeneous group, with large differences in fitness, comorbidities, and socioeconomic status. Consequently, older patients may experience very different levels of decline in physical, cognitive, and psychological functioning after breast cancer diagnosis and its related treatment.^2,3^

Previous research has shown the potential of physical activity to improve psychological outcomes, body composition, and quality of life.^4^ Furthermore, physical activity plays an important role in the prevention of other health problems in older patients, such as cardiovascular diseases, hypertension, obesity, and osteoporosis. These conditions may ultimately contribute to decreased levels of physical activity and an increased need for assistance with activities of daily living (ADL) and instrumental activities of daily living (IADL). Therefore, it is important to maintain physical activity levels and ADL/IADL independency after breast cancer diagnosis and treatment.

The aim of the current study was to assess changes in physical activity and ADL/IADL dependency in the first 5 years after breast cancer diagnosis in a real-world cohort of older women with in situ or stages I-III breast cancer and to investigate whether geriatric characteristics, breast cancer treatment, quality of life, depression, apathy, and loneliness were associated with these changes.

## Materials and Methods

### Study Design and Participants

This study used data from the prospective, multicenter observational Climb Every Mountain study, which has been previously described in detail.^5,6^ In short, patients aged 70 years and older who underwent surgery for primary, in situ or stages I-III breast cancer were recruited from 9 Dutch hospitals between 2013 and 2018. At baseline, patients underwent a geriatric assessment as part of standard care and they were followed up at 3, 9, 15, 27, and 60 months after surgery. Participants were included in this paper if questionnaires on both physical activity and ADL/IADL were available at baseline and at least at one other time point during follow-up. Written informed consent was obtained from all participants and the study was approved by the medical ethics committee of the Leiden University Medical Center.

### Data Collection

The baseline geriatric assessment included comorbidities prior to breast cancer diagnosis (Charlson Comorbidity Index (CCI)),^7^ medication use, cognition (Mini Mental State Examination (MMSE)), ADL/IADL (Groningen Activity Restriction Scale (GARS)),^8^ and the Timed Up and Go test.^9^ The CCI is a method of categorizing comorbidities of patients to predict 10-year survival rates (range 0-33). A higher CCI reflects more comorbidities. The MMSE ranges from 0 to 30, with higher scores indicating better cognitive functioning. ADL/IADL were assessed with the GARS questionnaire. The GARS contains 18 questions, of which 11 items are about ADL and 7 about IADL. The total score ranges from 18 to 72. The GARS was categorized into 4 groups (<19: no dependency, 19-28: some dependency, ≥29: disabled, and unknown).^10^ If less than 10% of the answers were missing per individual (ie, only one question), the average of the other answers was taken and recorded. If more than 10% of all answers in an independent questionnaire were missing, the whole questionnaire was classified as unknown. The TUG test measures the time that patients need to get up from a chair, walk a distance of 3 meters, turn around, and sit down again. This was done 3 times and the average score was used for the analyses. The cutoff point for normal mobility is ≤12 seconds.^11^ Trained personnel collected clinical data including patient-, tumor-, and treatment characteristics from medical records. Follow-up consisted of multiple assessments and questionnaires, including quality of life (EORTC QLQ-C30 and EORTC QLQ-BR23),^12,13^ the Cantril Ladder (range 0-10) for overall patient satisfaction,^14^ depression (15-item Geriatric Depression Scale),^15^ loneliness (De Jong Gierveld Loneliness Scale),^16^ apathy (Starkstein Apathy Scale),^17^ ADL/IADL (GARS) and physical activity. For the EORTC QLQ-BR23 questionnaire, the optional questions about sexual function, sexual enjoyment, and upset by hair loss were excluded from the total score, because these questions were answered by a limited number of patients. The 15-item Geriatric Depression Scale is a shortened questionnaire to assess depression in older adults. The total score ranges from 0 to 15 and higher scores indicate more depressive symptoms. Loneliness scores add up to a score between 0 and 11 with higher scores reflecting more severe loneliness. The Starkstein Apathy Scale consists of 14 questions with a score ranging from 0 to 42 with higher scores indicating greater apathy. ADL/IADL were assessed with the GARS questionnaire at baseline, 3, 9, 15, 27, and 60 months after diagnosis. Physical activity was assessed according to the Nurses’ Health Study II Activity and Inactivity Questionnaire for Metabolic Equivalent of Task (MET) Hours at 15, 27, and 60 months after diagnosis.^18^ At 3 months after diagnosis, patients were asked to record prediagnostic physical activity. In this questionnaire, patients indicate the average frequency of varying activities in that year, ranging from household activities to vigorous sports. MET-hours per week were calculated by multiplying the average hours per week spent at each activity with its specific intensity score based on the updated Physical Activity Compendium.^19^ Total physical activity per person was calculated by summing up all MET-hours per activity in each individual.

### Statistical Analyses

Baseline characteristics of patients with missing scores on the GARS or physical activity questionnaires at follow-up (15, 27, and 60 months) were compared with patients without missing data using the chi-square test. Changes in ADL/IADL and physical activity from baseline were evaluated for minimal clinically important differences. Based on previously reported cutoffs, a mean change of 3 MET-hours per week was considered as clinically relevant.^20^ No clinically significant cutoff has been determined for the GARS questionnaire.^21^ Linear mixed models were estimated to assess the longitudinal behavior of physical activity and ADL/IADL. Baseline characteristics of age, tumor stage, most extensive breast surgery, adjuvant systemic therapy, CCI, and BMI were incorporated into the models. To study whether longitudinal changes differed for both outcome variables (ADL/IADL and physical activity) per age group or GARS at baseline, interaction terms between time and age or GARS at baseline were included in the model. Linear mixed models were also used to analyze associations between variables assessed at 3 months post-diagnosis (Timed Up and Go test, quality of life, depression, apathy, and loneliness) and changes in ADL/IADL or physical activity between 15 and 60 months after diagnosis. The significance threshold for all analyses was set to an alpha of 0.05, and analyses were performed in IBM SPSS Statistics version 25.

## Results

A total of 239 patients were included (Table 1). The median age was 74 years (interquartile range (IQR) 72-78 years). Of all patients, 93 (39%) had a CCI of one or more and 117 (49%) patients were not fully independent in their ADL and IADL. Most patients were diagnosed with stage I or II breast cancer. Ninety-eight (41%) patients were treated with a mastectomy and 141 (59%) patients with breast conserving surgery. A total of 154 (65%) patients received postoperative radiotherapy. Around half of all patients received adjuvant systemic treatment, of which 109 (46%) patients endocrine therapy, 8 (3%) chemotherapy, and 8 (3%) a combination of both.

**Table 1.** Patient-, tumor-, and treatment characteristics at baseline.

	*N*	%
Age, years		
70-74	132	55.2
75-79	54	22.6
≥80	53	22.2
Charlson Comorbidity Index (CCI)		
0	146	61.1
1	52	21.8
≥2	41	17.1
Polypharmacy		
No	147	61.5
Yes	80	33.5
Unknown	12	5.0
BMI		
20-24.9	78	32.6
<20	11	4.6
≥25	150	62.8
Functional status (GARS)^a^		
<19: no dependency	122	51.0
19-28: some dependency	105	44.0
≥29: disabled	12	5.0
MET-hours/week^b^		
Continuous (median (IQR))	23 (8-55)	
Stage		
0	10	4.2
I	122	51.0
II	78	32.6
III	14	5.9
Unknown	15	6.3
Grade		
I	55	23.0
II	105	44.0
III	72	30.1
Unknown	7	2.9
Most extensive surgery		
Breast conserving	141	59.0
Mastectomy	98	41.0
Most extensive axillary surgery		
No axillary surgery	13	5.4
Sentinel lymph node dissection	182	76.2
Axillary lymph node dissection	44	18.4
Adjuvant systemic treatment		
No systemic adjuvant treatment	114	47.7
Endocrine therapy (ET)	109	45.7
Chemotherapy (CT)	8	3.3
Combination of ET and CT	8	3.3
Adjuvant radiotherapy		
No	85	35.6
Yes	154	64.4

^a^Higher scores on the GARS questionnaire indicate a worse functional status, range 18-72.

^b^Higher numbers of MET-hours per week indicate more physical activity, range 0-∞.

Abbreviations: BMI, body mass index; CCI, Charlson Comorbidity Index; CT, chemotherapy; ET, endocrine therapy; GARS, Groningen Activity Restriction Scale; IQR, interquartile range; MET, metabolic equivalent of task.

The response rates to the questionnaires were 93%, 96%, and 88% at 15, 27, and 60 months after diagnosis, respectively (Supplementary Fig. S1). At 60 months of follow-up, patients with missing data were statistically significantly older than patients without missing questionnaires. CCI, polypharmacy, GARS at baseline, tumor stage, and adjuvant systemic treatment were similar for these 2 groups at 15, 27, and 60 months follow-up.

**Figure 1.**
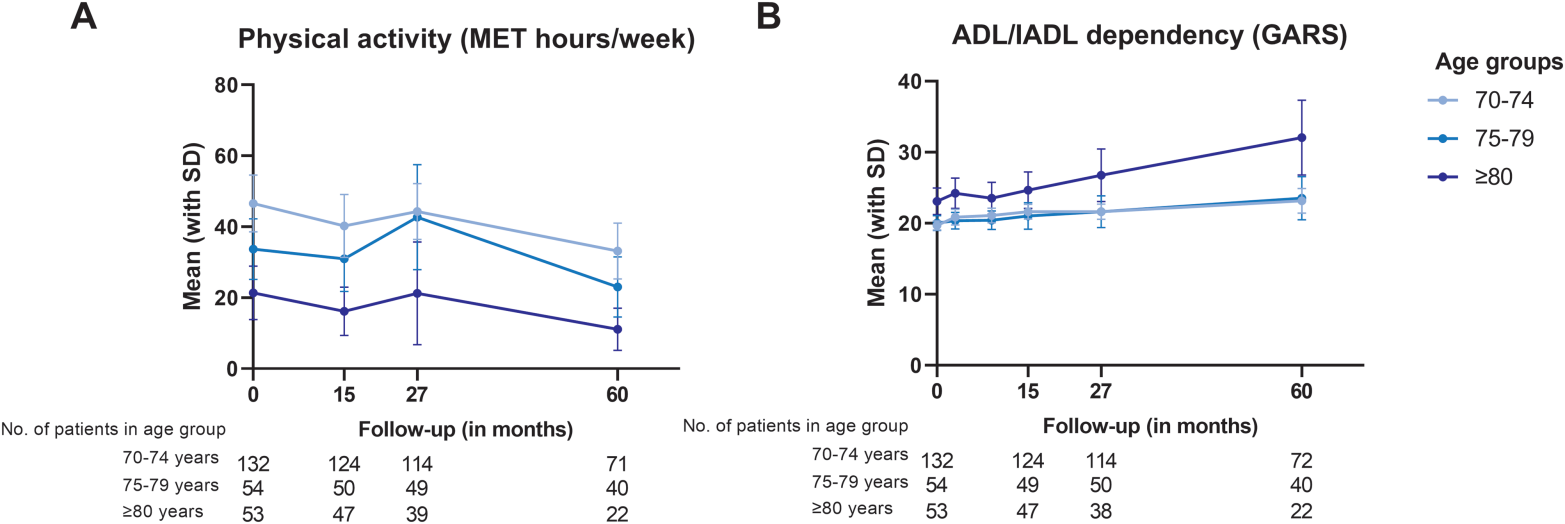
Physical activity (**A**) and ADL/IADL dependency (**B**) during 5-year follow-up.

Mean values for physical activity measured in MET-hours per week at baseline, 15, 27, and 60 months after diagnosis are shown in Fig. 1A. In the first 5 years after diagnosis, physical activity decreased in all age groups, and was lowest for patients aged 80 years and older in a multivariate model (Table 2). Mean changes over 5-year follow-up were clinically relevant for all age groups (age 70-74: −13.4; 75-79: −10.6; ≥80: −10.2). Patients with dependencies in ADL/IADL at baseline remained less active over the first 5 years after breast cancer diagnosis (GARS ≥19: *β* = −9.68, 95% CI, −15.61 to −3.76, *P* = .001, compared to GARS <19), but the longitudinal change was not statistically significantly different from patients who were independent in their ADL/IADL at baseline. Moreover, patients with a CCI of 1 and more had lower levels of physical activity over time. Type of surgery and adjuvant systemic therapy were not associated with physical activity.

**Table 2.** Changes in physical activity (MET-hours/week) and ADL/IADL (GARS) during 5-year follow-up, multivariate linear mixed model.

	MET-hours/week^a^			GARS^b^		
	β	95% CI	*P*-value	β	95% CI	*P*-value
Age, years						
70-74	Reference			Reference		
75-79	−8.44	−15.11 to −1.76	.013	−0.61	−1.46 to 0.25	.166
≥80	−20.37	−28.22 to −12.52	<.001	1.91	0.89 to 2.92	<.001
Stage						
0-I	Reference			Reference		
II	−2.14	−9.29 to 5.01	.556	0.29	−0.62 to 1.19	.534
III	−12.35	−24.83 to 0.14	.053	0.62	−0.95 to 2.20	.437
Unknown	15.00	3.40 to 26.61	.011	-1.80	−3.26 to −0.34	.016
Most extensive breast surgery						
Breast conserving	Reference			Reference		
Mastectomy	4.17	−1.81 to 10.16	.171	0.09	−0.67 to 0.85	.823
Adjuvant systemic therapy						
No	Reference			Reference		
Yes	−1.03	−7.14 to 5.09	.742	−0.51	−1.28 to 0.26	.195
Charlson Comorbidity Index (CCI)						
0	Reference			Reference		
1	−15.12	−22.09 to −8.14	<.001	2.22	1.33 to 3.10	<.001
≥ 2	−11.50	−19.14 to −3.87	.003	3.02	2.05 to 3.99	<.001
BMI						
20-24.9	Reference			Reference		
<20	−13.72	−27.25 to −0.20	.047	0.40	−1.33 to 2.13	.651
≥25	−8.78	−14.80 to −2.77	.004	1.06	0.28 to 1.83	.008
GARS at baseline						
<19	Reference			Reference		
≥19	−9.68	−15.61 to −3.76	.001	3.51	2.76 to 4.26	<.001
MET-hours/week at baseline						
Continuous	N/A	N/A	N/A	−0.02	−0.03 to −0.01	<.001

^a^Higher numbers of MET-hours per week indicate more physical activity, range 0-∞.

^b^Higher scores on the GARS questionnaire indicate a worse functional status, range 18-72.

Intercept of MET-hours model was 56.48.

Intercept of GARS model was 19.17.

Abbreviations: ADL, activities of daily living; BMI, body mass index; CCI, Charlson Comorbidity Index; GARS, Groningen Activity Restriction Scale; IADL, instrumental activities of daily living; MET, metabolic equivalent of task.

The GARS gradually increased over time with a relatively stronger increase for patients aged 80 years and older (Fig. 1B). Patients with dependencies in ADL/IADL at baseline experienced further decline over time in a multivariate model (Table 2), but with a similar longitudinal change to those who were fully independent in their ADL/IADL at baseline. A higher level of physical activity in MET-hours per week at baseline was associated with a small, but statistically significant better GARS during follow-up (*β* = −0.02, 95% CI, −0.03 to −0.01, *P* < .001). Patients with a CCI of ≥2 developed more deficiencies in ADL/IADL over time (*β* = 3.02, 95% CI, 2.05-3.99, *P* < .001) when compared to patients without comorbidities according to the CCI. Type of surgery and adjuvant systemic therapy were not associated with changes in ADL/IADL.

Linear mixed models were estimated to investigate whether the Timed Up and Go test, quality of life, depression, apathy, and loneliness at 3 months after diagnosis were associated with changes in physical activity and ADL/IADL between 15 and 60 months after diagnosis (Table 3). Quality of life was associated with physical activity after adjustment for confounders: every point increase on the generic or breast cancer-specific quality of life questionnaires was associated with a longitudinal increase of 0.8 and 0.5 MET-hour per week, respectively (*P* < .001). A higher life satisfaction, as assessed by the Cantril Ladder, was associated with greater physical activity levels (*β* = 6.91, 95% CI, 4.02-9.79, *P* < .001). Patients with increasing depression or loneliness symptoms at 3 months follow-up were less physically active over time.

**Table 3.** Association between assessments/questionnaires assessed at 3 months follow-up and physical activity (MET-hours/week) and ADL/IADL (GARS) between 15 and 60 months follow-up, univariate linear mixed model.^a^

	MET-hours/week^b^			GARS^c^		
	β	95% CI	*P*-value	β	95% CI	*P*-value
Timed up and go test						
<12 sec	Reference			Reference		
>12 sec	0.50	−10.61 to 11.61	.930	5.36	3.34 to 7.39	<.001
Generic quality of life (EORTC QLQ-C30)^d^						
Continuous	0.80	0.47 to 1.12	<.001	−0.25	−0.31 to −0.20	<.001
Breast cancer-specific quality of life (EORTC QLQ-BR23)^d^						
Continuous	0.52	0.25 to 0.79	<.001	−0.19	−0.24 to −0.15	<.001
Life satisfaction (Cantril Ladder)^d^						
Continuous	6.91	4.02 to 9.79	<.001	−1.51	−2.04 to −0.99	<.001
Geriatric Depression Scale^e^						
Continuous	−3.59	−5.18 to −2.01	<.001	1.10	0.82 to 1.38	<.001
Starkstein Apathy Scale^e^						
Continuous	−0.43	−1.14 to 0.28	.236	0.12	−0.01 to 0.24	.077
De Jong Gierveld Loneliness Scale^e^						
Continuous	−1.59	−3.01 to −0.17	.028	0.36	0.10 to 0.62	.007

^a^All variables are adjusted for age, tumor stage, most extensive breast surgery, adjuvant systemic therapy, Charlson Comorbidity Index, and BMI. Unknown values are not included in this table.

^b^Higher numbers of MET-hours per week indicate more physical activity, range 0-∞.

^c^Higher scores on the GARS questionnaire indicate a worse functional status, range 18-72.

^d^Higher scores indicate a better quality of life/life satisfaction.

^e^Higher scores indicate greater symptoms.

Abbreviations: ADL, activities of daily living; GARS, Groningen Activity Restriction Scale; IADL, instrumental activities of daily living; MET, metabolic equivalent of task.

Similar results were seen for changes in ADL/IADL between 15 and 60 months follow-up (Table 3), in which a better quality of life was associated with better preservation of ADL/IADL (EORTC QLQ-C30: *β* = −0.25, 95% CI, −0.31 to −0.20, *P* < .001; EORTC QLQ-BR23: *β* = −0.19, 95% CI, −0.24 to −0.15, *P* < .001; Cantril Ladder: *β* = −1.51, 95% CI, −2.04 to −0.99, *P* < .001). Depression and loneliness scores after 3 months post-diagnosis were associated with an increase in dependency during follow-up.

## Discussion

This real-world cohort study of patients aged 70 years and older with breast cancer showed a small decline in physical activity and a small increase in dependency in the first 5 years after diagnosis. Physical activity in each age group at the end of follow-up was similar to baseline levels of the older age groups, which implies a natural course of aging. Geriatric characteristics at baseline (ie, age, comorbidities, BMI, and GARS) were strongly associated with longitudinal change in ADL/IADL dependency and physical activity, whereas breast cancer characteristics and treatment were not. Moreover, after completion of locoregional treatment, quality of life, depression, and loneliness were associated with changes in physical activity and ADL/IADL dependency during 5-year follow-up.

Although changes in ADL/IADL and physical activity were small, it is important to assess these parameters in the older population as they could interfere with independent living. Older patients may value quality of life and functional independence over other treatment outcomes, such as recurrence and survival.^22^ The deterioration of ADL/IADL and physical activity is mainly age-related. Nevertheless, our results show a significant association between depressive symptoms and loneliness with both ADL/IADL dependency and physical activity. Depressive symptoms have previously been linked with impaired ADL/IADL in patients with breast cancer, but the studies had a short follow-up period and did not specifically focus on older patients.^23,24^ Since association is different from causation, it is unclear whether reduced physical activity and dependency are a consequence of depressive symptoms or rather a cause. Nevertheless, our results in an older population with a relatively long follow-up support the need for early detection of psychological disorders and incorporation of not only exercise interventions but also psychological interventions into breast cancer care for older patients. Previous meta-analyses investigated the effectiveness of specific psychological interventions in women with breast cancer and showed that individually delivered cognitive behavioural therapy effectively reduces depressive symptoms.^25,26^ However, the meta-analyses included studies with several limitations and none to very few older patients. The same applies to studies on specific intervention programs for loneliness.^27-29^ Further research is required to identify effective intervention strategies for older patients with breast cancer.

The association between geriatric characteristics, rather than the association between cancer-specific variables and changes in ADL/IADL is in line with previous studies. A study including nearly 6000 nursing-home residents from the US, found a higher ADL score (signifying greater dependency) in more than half of all patients one year after breast cancer surgery.^30^ In contrast, in 2 studies with younger and fitter women, a fifth of patients had a functional decline at one-year follow-up.^31,32^ Another study focusing specifically on relatively fit older patients treated with chemotherapy demonstrated that 30% had a functional decline 1 year after chemotherapy initiation.^33^ However, all 4 studies assessed functional decline during a short time-window. The Age Gap observational cohort study into 3300 women aged 70 years and older with breast cancer assessed ADL in the first 24 months after diagnosis.^34^ They found that patients who received surgery had an early decrease in functional status between baseline and 6 weeks which failed to recover to baseline levels at 24 months follow-up, while patients treated with primary endocrine therapy had a more gradual decline. In this study, functional status was assessed with one question while our study used a complete questionnaire. Nevertheless, our study shows a similar pattern in physical function in the first 27 months of follow-up but additionally shows that the decline in physical function continues over the subsequent 36 months, especially in the oldest age group. The Tamoxifen Exemestane Adjuvant Multinational (TEAM) trial investigated functional decline and physical activity in both younger and older patients and the authors found that patients of all age groups who were treated with surgery and adjuvant endocrine therapy became less physically active in the first 2 years after diagnosis.^2^ Older patients did not fully recover to their pre-diagnostic independency levels in the first 2 years, while younger patients did. This study only included relatively fit older patients with an Eastern Cooperative Oncology Group performance status of zero or one and the questionnaire on a physical dependency was limited. Finally, a cohort study by Huy et al assessed physical activity one year after breast cancer surgery by including both young (aged < 65 years) and old patients.^35^ The authors showed a median decrease of 4 MET-hours per week one year after surgery in older patients, while younger patients showed a median increase of 2.2 MET-hours per week.

The most important strength of this study is the longitudinal design with detailed information on older patients with a long follow-up period and a high response rate (88%-96%). There are also limitations to this research. Although the aim of the study was to present a real-time cohort, it only included patients whose questionnaires on both physical activity and ADL/IADL were available at baseline and at least at one other time point during follow-up, resulting in a relatively healthy older population. Another limitation is that recall bias might exist in measuring baseline physical activity as this was assessed 3 months after diagnosis. Nevertheless, all other questionnaires were examined prospectively. In addition, physical activity was not objectively assessed via accelerometers. However, a previous study showed that patient-reported physical activity is concordant with more objective accelerometers.^36^ Finally, this study did not include a control group to compare the observed physical decline with patients without breast cancer. However, our research group is currently comparing the GARS questionnaire of older patients diagnosed with breast cancer with a similar cohort of older adults without breast cancer. Preliminary results confirm our findings and show no longitudinal differences in ADL/IADL between these cohorts.

## Conclusion

Patients aged 70 years and older with breast cancer showed a small decline in physical activity and a small increase in ADL/IADL dependency in the first 5 years after diagnosis. However, these changes did not seem to be related to breast cancer or its treatment, but rather to pre-existent geriatric characteristics, loneliness, and depressive symptoms. These findings may help to provide patients and their caregivers with additional information to reassure them that in older patients with breast cancer, the long-term effects of breast cancer and its treatment on physical activity and ADL/IADL dependency are likely to be minimal.

## Supplementary Material

oyad027_suppl_Supplementary_Figure_S1

## Data Availability

The data underlying this article will be shared on reasonable request to the corresponding author.
