# Supplementary material for: Physical Function and Physical Activity in Older Breast Cancer Survivors: 5-Year Follow-Up from the Climb Every Mountain Study
Source: Oncologist. 2023 Mar 21;28(6):e317–23. doi: 10.1093/oncolo/oyad027 (PMC10243764; doi:10.1093/oncolo/oyad027)
Supplement: oyad027_suppl_Supplementary_Figure_S1 [file oyad027_suppl_supplementary_figure_s1.docx]

**CLIMB patients aged ≥ 70 years who underwent surgery for breast cancer**

*N* = 354

**Total participants**

*N* = 239

**Baseline**

**Follow-up questionnaire**

Received *N* = 239

Completed *N* = 223

**15 months**

**60 months**

**27 months**

**Follow-up questionnaire**

Received *N* = 214

Completed *N* = 206

**Follow-up questionnaire**

Received *N* = 153

Completed *N* = 134

**Excluded**

No questionnaire available at baseline or at ≥ 1 other time point during follow-up

*N =*115

**Excluded**

Withdrawal of consent *N* = 14

Loss to follow-up *N* = 6

Deceased *N =* 5

**Excluded**

Less than 5 years follow-up *N =* 36

Withdrawal of consent *N* = 3

Loss to follow-up *N* = 1

Deceased *N =* 21

**Supplemental Figure 1:** Flowchart
